# Short-term Prediction of the Incidence of Congenital Rubella Syndrome

**DOI:** 10.1371/currents.outbreaks.8c74272f4348781c5d01c81e6150c2f7

**Published:** 2014-10-30

**Authors:** Yasushi Ohkusa, Tamie Sugawara, Satoru Arai, Hiroshi Satoh, Hideo Okuno, Keiko Tanaka-Taya, Kazunori Oishi

**Affiliations:** Infectious Disease Surveillance Center, National Institute of Infectious Diseases, Shinjuku, Tokyo, Japan; Infectious Disease Surveillance Center, National Institute of Infectious Diseases, Shinjuku, Tokyo, Japan; Infectious Disease Surveillance Center, National Institute of Infectious Diseases, Shinjuku, Tokyo, Japan; Infectious Disease Surveillance Center, National Institute of Infectious Diseases, Shinjuku, Tokyo, Japan; Infectious Disease Surveillance Center, National Institute of Infectious Diseases, Shinjuku, Tokyo, Japan; Infectious Disease Surveillance Center, National Institute of Infectious Diseases, Shinjuku, Tokyo, Japan; Infectious Disease Surveillance Center, National Institute of Infectious Diseases, Shinjuku, Tokyo, Japan

**Keywords:** congenital rubella syndrome, public health policy, rubella, short-term prediction, Surveillance

## Abstract

Objectives In Japan, a rubella outbreak occurred from early 2012 to late 2013, primarily among adult males aged 20–49 years. We conducted this study to predict the number of congenital rubella syndrome (CRS) cases in Japan in 2014. Methods The probability of CRS when a pregnant woman is infected with rubella depends on the gestational age of the fetus. The cumulative number of CRS cases was predicted by a formula based on the parameters from two studies conducted in the U.K. and the U.S., the reported cases of rubella among women 15–49 years of age, and the reports of CRS from 2011 to week 2 of 2014. Findings While the predicted number of cases of CRS based on parameters from the U.K. study demonstrated a biphasic curve, with a low peak around week 12 and a high peak around week 50 of 2013, the predicted number of CRS cases based on the U.S. study demonstrated a single peak around week 50 of 2013. The ex post evaluation indicated that the cumulative number of CRS cases in 2014 would be 19.1–29.3. Interpretation Our prediction of the number of CRS cases may be useful for the enhanced detection of this often under-reported syndrome.

## Introduction

Rubella is usually a mild, febrile illness in children and adults, and up to 50% of rubella infections are asymptomatic. However, maternal infection with rubella, especially in early pregnancy, can cause an infant to be born with severe birth defects that are known as congenital rubella syndrome (CRS) 1 .

In 1976, Japan introduced single-antigen rubella vaccine in it’s national immunization program, targeting girls in junior high school. In 1989, measles-mumps-rubella (MMR) vaccine was introduced, targeting children aged 12-72 months. The MMR vaccine was, however, interrupted because of a high incidence of aseptic meningitis due to mumps vaccine. Although Japan introduced single-antigen rubella vaccine targeting children aged 12-90 months and boys and girls in junior high school in 1995, the coverage of this vaccine was low. A recent study reported that seropositivity for rubella antibody (≧1:8) among adults aged 30-50 years was 73%-86% for males and 97%-98% for females 2 . These data suggest that adult males remain susceptible to rubella.

A nationwide, sentinel case-based surveillance for CRS was established in April 1999. The sentinel surveillance systems were replaced by nationwide case-based surveillance for rubella in January 2008, and all physicians were required to report any clinically diagnosed or laboratory-confirmed rubella case to local public health offices.

The accumulated unvaccinated population led to an outbreak of rubella, primarily among adult males aged 20–49 years, from early 2012 to late 2013 3 . This epidemic of rubella peaked between weeks 19 and 22 of 2013. Because an epidemic of CRS follows a rubella epidemic with a lag of approximately 6–7 months in Greece 4 , an increase in CRS cases might occur from late 2013 to early 2014. The Ministry of Health, Labour and Welfare recommended not only routine immunization for children one and six years of age as part of the national immunization programme, but also encouraged vaccination of family members of pregnant women and vaccination for women who planned to get pregnant, as a counter-measure to reduce the rubella epidemic and the incidence of CRS 3 .

Although it is important from a public health viewpoint to predict the number of CRS cases following a rubella epidemic, no studies have been conducted to predict the number of CRS cases during the period of a rubella epidemic. The aim of this study was to predict the number of CRS cases in 2014 in Japan using a formula for predicting CRS cases based on the parameters of two studies conducted in the U.K. and the U.S.

## Materials and Methods

The risk of CRS has previously 5 been calculated using :1) the number of rubella cases in women of childbearing age, 2) the proportion of pregnancies, and 3) the CRS risk based on gestational age.

1) The number of rubella cases in women of childbearing age

This parameter was obtained from the epidemic curve of rubella cases. Childbearing age was defined as 15–49 years of age.

2) The proportion of pregnancies

The proportion of pregnancies was obtained by dividing the number of deliveries divided by the population of women in each age group. In 2010, the numbers of births were 13,494, 110,956, 306,913, 384,382, 220,103, 34,610, and 773 for mothers aged 15–19, 20–24, 25–29, 30–34, 35–39, 40–44, and 45–49 years, respectively. The total population of women in each age group was (in thousands) 2,954, 3,160, 3,602, 4,120, 4,836, 4,341, and 4,005, respectively, in the same year 6 .

3) The CRS risk based on gestational age

Gestational age was assumed to be independent of rubella virus infection, i.e., the gestational age at which a pregnant woman became infected with the rubella virus would be distributed uniformly. The probability of infection for each gestational week was assumed to be 1/39, as the duration of pregnancy can be defined at 2–40 gestational weeks. The probability of CRS depending on the gestational age of the fetus when the pregnant woman acquires rubella virus infection 7 was calculated from two studies conducted in the U.K. 8 and the U.S. 9 (Table 1).

**Table 1: Congenital Rubella Syndrome incidence (%) based on the fetal gestational age in pregnant women with rubella virus infection:** 

	U.K.study					U.S. study				
Gestational age (weeks)	≤10	11-12	13-14	15-16	≥17	≤4	5-8	9-12	13-16	≥17
CRS incidence (%)	90	33	11	24	0	70	40	25	40	8

Table 1 Note: Data are from studies conducted in the U.K. 8 and the U.S. 9 .

These three components suggest that the expected number of CRS cases during period *t* should be \begin{equation*}\sum_{i=1}^{n}{Prob(\text{CRS of }i\text{ at }t) }\end{equation*}, where *n* is the total number of rubella-infected women, which represents the magnitude of the ongoing rubella outbreak, and \begin{equation*}{Prob(\text{CRS of }i\text{ at }t) }\end{equation*} is defined as , \begin{equation*}q\left( a\left( i \right)  \right) \frac{1}{39} p\left( t-r\left( i \right)  \right) \end{equation*} where *a(i)* is age of patient *i*, which represents the age distribution of female rubella patients with age 15–49, *q*(･) is the probability of pregnancy by age *i*, *p*(･) is the probability of CRS by gestational age when pregnant and rubella infected and \begin{equation*}p\left( \cdot  \right) =0 \text{    } if \text{ } t-r\left( i \right) \geq 40\end{equation*},and *r*(*i*) is the date of implantation of *i*, where \begin{equation*}t-r\left( i \right) \end{equation*} is gestational age.

Hereafter, \begin{equation*}\sum_{i=1}^{n}{Prob(\text{CRS of }i\text{ at }t) }\end{equation*} is referred to as the CRS potential. Therefore, the CRS potential is defined as the theoretical predicted number of CRS cases based on a rubella outbreak in women. However, the CRS potential might not be equal to the number of reported CRS cases because of under-reporting of rubella and CRS cases or asymptomatic cases. To bridge these gaps, we regressed the number of reported CRS cases based on the CRS potential at birth, \begin{equation*}\left( CRS \text{ }case \right) _{t}=\alpha +\beta \left( CRS \text{ } potential \right)_{t} \end{equation*}, by the ordinary least squares method, which is the simplest regression procedure, in which parameters are estimated to minimize the sum of the square of the residual. Data period for estimation was between week 1, 2011 and week 40, 2013.

Finally, to verify the precision of prediction, we performed an ex post evaluation, which is an evaluation of the future based on data available at the time of estimation. Thus, we used data only up to week 40 of 2013 to predict up to week 20of 2014, and compared this with the actual CRS incidence from week 41, 2013 to week 20, 2014.

## Results

Following a relatively small outbreak in 2012, a larger outbreak occurred in 2013 (Figure 1). Until week 20, 2014, 3021 rubella cases occurred among women 15–49 years old and 44 CRS cases were reported in accordance with the Infectious Disease Law. The numbers of rubella cases and CRS cases were peaked at week 19, 2013 and at week 2, 2014, respectively. While the CRS potential based on the U.K. study demonstrated a biphasic curve with a low peak around week 12 and a high peak around week 50 of 2013 (Figure 2), the CRS potential based on the U.S. study demonstrated a single peak around week 50 of 2013.

**The number of reported rubella cases (blue line) in women 15–49 years old and congenital rubella syndrome (CRS) cases (red bar) in Japan between week 1, 2011 and week 2, 2014.**
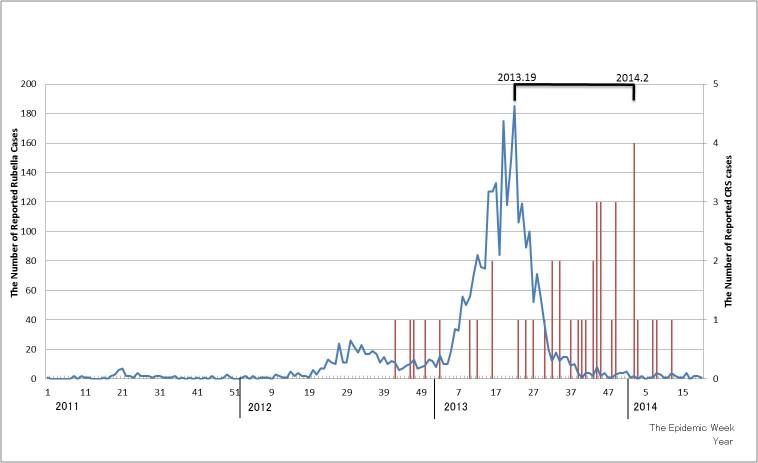

**The congenital rubella syndrome (CRS) potential based on the studies conducted in the U.K. (black line) and the U.S. (green line).**
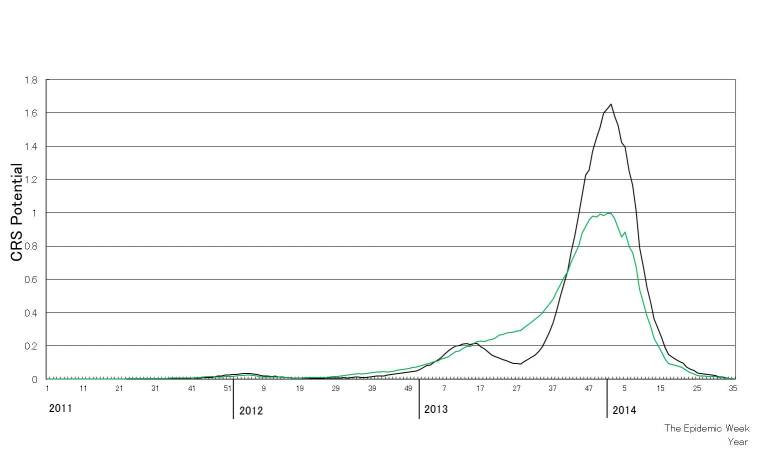

The estimated parameters for the predicted number of CRS cases are shown in Table 2. The estimated b was 0.99 based on the U.K. study and 1.46 based on the U.S. study. The estimated bs indicate that the number of CRS cases is the same as the CRS potential or 1.5 times greater than the CRS potential.Table 2: Estimation of parameters for the predicted number of Congenital Rubella Syndrome cases.

**Table 2: Estimation of parameters for the predicted number of Congenital Rubella Syndrome cases:** 

		Estimated coefficient	*t*-value	*p*-value
U.K.	α	0.0821	1.80	0.073
	β	0.993	8.77	0.000
U.S.	α	0.0158	0.34	0.735
	β	1.463	9.69	0.000

Table 2 Note: The determinant coefficients, an appropriate index for a fitness of the estimation, of both estimations are 0.330 for the upper panel and 0.376 for the lower panel. Data period for estimation was since week 1,2011 to week 40, 2013.

Figure 3 shows the predicted value, the estimated α + the estimated β (CRS potential), during the period covered by the data used for estimation, which allows prediction of the actual data before week 2, 2014. The predicted cumulative number of CRS cases based on the U.K. and U.S. studies is similar, and both estimations provide a close fit with the actual cumulative number of CRS cases.

**Comparison between the actual cumulative number of congenital rubella syndrome (CRS) cases (red line) and the predicted cumulative number of CRS cases. The black line and green line are the predicted cumulative numbers of CRS cases based on the studies conducted in the U.K. and the U.S., respectively, using the data up to week 40, 2013.**
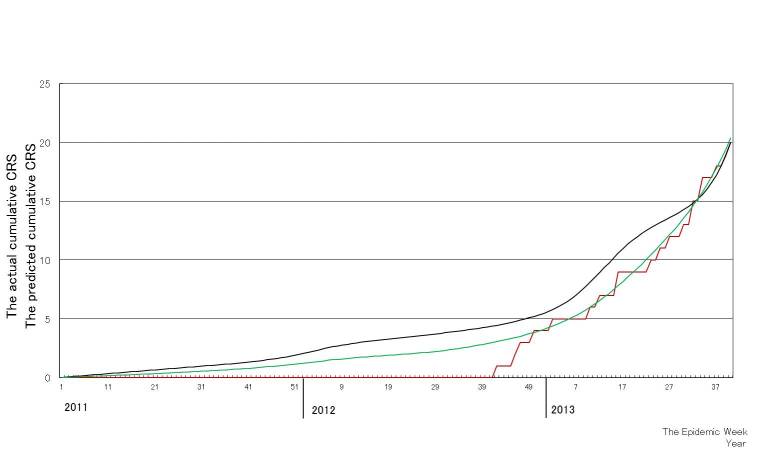

The cumulative number of CRS cases in the future at the time of prediction, i.e., the period after week 2, 2014, is shown in Figure 3. The cumulative number of CRS cases in 2014 is predicted to be 19.1–29.3.

The results of the ex post evaluation are shown in Figure 4. The curve of the predicted cumulative number of CRS cases based on the U.S. data is**close to**the curve of the actual cumulative number of CRS cases between week 40, 2012 and week 40, 2013, while the curve of the predicted cumulative number of CRS cases based on the U.K. data is slightly higher than the curve of the actual cumulative number of CRS cases during the same period. After week 47, 2013, the curve of the predicted cumulative number of CRS cases based on the U.S. data is close to the actual cumulative number of CRS cases** until around week 5, 2014. **In contrast, the curve of the predicted cumulative number of CRS cases based on the U.K. data is much higher than the curve of the actual cumulative number of CRS cases.

**Ex post evaluation of the predicted cumulative numbers of congenital rubella syndrome (CRS) cases. The red line indicates the actual cumulative number of CRS cases up to week 2, 2014. The black line and green line are the predicted cumulative numbers of CRS cases based on the studies conducted in the U.K. and the U.S., respectively, using the data up to week 40, 2013.**
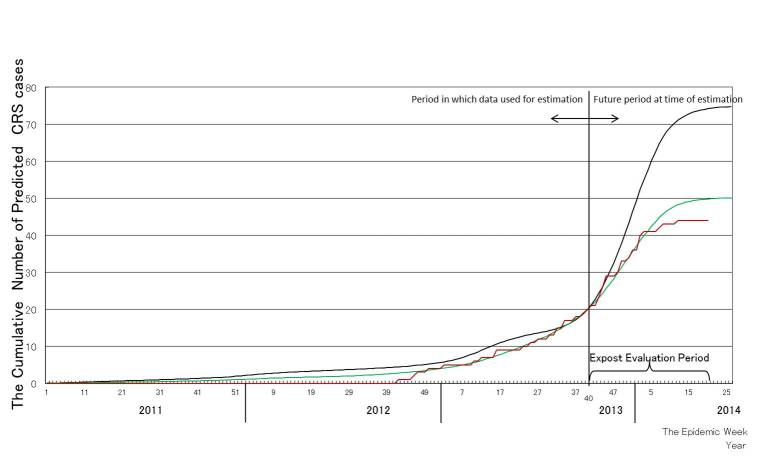

## Discussion

In this study, we predicted the number of CRS cases with a formulation based on the parameters from two studies from the U.K. and the U.S. in 2013 and up to week 25, 2014. We found an increase of CRS cases following the rubella epidemic with a lag of 5-7 months, which was almost in agreement with a previous experience in Greece 4 . A close correlation was found between the predicted cumulative number of CRS cases in 2013 and the actual cumulative number of reported CRS cases, validating the present method of CRS prediction (Figure 3). The ex post evaluation generated two different patterns of the predicted number of CRS cases: a higher prediction based on parameters from the U.K. study and a lower prediction based on the U.S. study (Figure 4). The lower prediction based on the data from the U.S. agreed closely with the actual cumulative number of CRS cases. A dissociation between the predicted number of CRS cases based on two studies from the U.K. and the U.S. may be attributable to a difference in the probability of CRS, which directly affects the CRS potential, during the period up to 10 weeks of gestational age. While a higher percentage (90%) of CRS was found during the period up to 10 weeks of gestational age in the U.K. study, relatively lower percentages (40% or 70%, respectively) of CRS, compared with that in the UK study, during the period up to 10 weeks or between 5 and 8 weeks of gestational age were observed in the U.S. study.However, the reasons for the difference of the CRS incidence between the US and UK epidemics remain uncertain from the two literatures 8 ^,^ 9 . Actually, the estimation of β based on the past data retrospectively and thus it may not reflect the current or future situation. However, as shown in Figure 3 and ex post evaluation, it was proved to have a quite preciseness for the prediction of CRS and it would be valuable for public health workers.

CRS is clinically confirmed if an infant has: 1) at least two of cataract, congenital glaucoma, congenital heart disease, hearing impairment, or pigmentary retinopathy; or 2) one of these complications, and one of purpura, splenomegaly, microcephaly, meningoencephalitis, radiolucent bone disease, or jaundice developed within 24 hours after birth 3 . A cataract, which is found in approximately one third of all CRS babies, is occasionally not observed until late infancy 10 . Sensorineural deafness is also the most common manifestation of CRS, and deafness is frequently overlooked in infancy. Therefore, the difficulties in the clinical diagnosis of CRS often cause a delayed notification of CRS cases, and temporarily decrease the number of reported CRS cases compared with that predicted by our method.

In summary, we predicted the cumulative number of CRS cases in 2014 by a formula based on the parameters of two studies from the U.K. and the U.S. Our method for prediction of the number of CRS cases may be useful for the enhanced detection of this syndrome that is often under-reported.

## Competing Interest

The authors have declared that no competing interests exist.

## Author Bio

YO is a senior scientist of the Infectious Disease Surveillance Center, National Institute of Infectious Diseases, Japan, and works in biostatistics, cost-effectiveness, and mathematical modelling.
